# Investigation on Fatigue Threshold Testing Methods in a Near Lamellar TiAl Alloy

**DOI:** 10.3390/ma12213487

**Published:** 2019-10-24

**Authors:** Shiyuan Wang, Hangyue Li, Paul Bowen

**Affiliations:** 1 College of Material Science and Technology, Nanjing University of Aeronautics and Astronautics, Nanjing 211106, China; sy.wang@nuaa.edu.cn; 2 School of Metallurgy and Materials, University of Birmingham, Birmingham B15 2TT, UK

**Keywords:** near lamellar γ-TiAl alloy, fatigue testing method, fatigue crack threshold, fatigue crack growth, load-increasing method

## Abstract

The effects of influential fatigue testing factors, including loading schemes (e.g., traditional load shedding and staircase load increasing), morphology of crack starters, and precracking approaches on the near-threshold fatigue crack growth behaviors for a near lamellar γ-TiAl alloy (Ti-45Al-2Mn-2Nb-1B) were investigated at room temperature and 650 °C. The results showed that the measured fatigue threshold values in lamellar γ-TiAl alloys are very sensitive to the applied testing procedures. For example, the staircase load-increasing method yielded smaller threshold values. When such a load-increasing method was used, the threshold values were measured either from a notch machined by electro-discharge machining or prepared by a compression–compression fatigue loading. Moreover, some differences could be seen with respect to the morphologies of the crack starters. Most of the above influences are associated with the brittle nature of the material and the characteristics of the lamellar microstructures, and closure effects are primarily induced by crack wake roughness or unbroken ligaments.

## 1. Introduction

γ-TiAl alloys are viable lightweight candidates to replace nickel-based superalloys for weight-saving purposes in aeroengines. In 2006, a TiAl alloy (Ti4822) was first used for commercial application by General Electric (GE) in the low-pressure turbine blades of the GEnx^TM^ engine, showing that the development of γ-TiAl-based alloys has entered a commoditization stage [1]. As aeroengine materials, γ-TiAl alloys are faced with inevitable vibrations as a result of rotation that can induce hazardous high-cycle fatigue issues in components. In the meantime, low ductility and low toughness aggravates the fatigue problems in the γ-TiAl-based alloys. Over the last few decades, many studies into their fatigue behaviors and fatigue threshold have been carried out with respect to the effects of microstructure, temperature, environment, loading conditions, and even crack morphologies. As the fatigue crack growth (FCG) curves of TiAl alloys are relative steep, there is a consensus that the damage tolerance design for γ-TiAl alloys should be primarily operated with their fatigue threshold, ΔK_th_, to avoid any fatigue crack initiation in service. 

Numerous studies have employed different fatigue testing methods to obtain the ΔK_th_ values and FCG curves of lamellar γ-TiAl alloys. A summary of some examples of the testing methods used for determining ΔK_th_ values in γ-TiAl alloys is shown in Table 1. As seen in Table 1, the most commonly used specimen geometry is a compact tension (CT) configuration together with tension–tension loading schemes. Additionally, Edwards [2] reviewed the progress in understanding high-cycle fatigue behaviours of TiAl alloys and reported that different loading strategies by altering the way of applying K (stress intensity factor) and R (stress ratio) values, such as ΔK-decreasing (load-shedding), ΔK-increasing, constant K_max_, and constant R-loading methods, can have effects either on ΔK_th_ or FCG or both as a result of different plastic zone sizes.

It has been widely noticed that different loading schemes can result in the discrepancy of ΔK_th_ values in ductile metallics [13,14,15]. Most of the load-shedding procedures were conducted according to the American Society of Testing Materials (ASTM) E647 standard, which was designed for ductile metals [16]. Kranenburg et al. [13] performed fatigue threshold tests on an aluminum alloy (AA5083-H321) through the ASTM method and compared the results to that obtained using a “constant K_max_, increasing K_min_” method. The ASTM method led to higher ΔK_th_ values because of the crack closure effect near the end of the tests. Additionally, the ΔK_th_ values obtained via the “constant K_max_, increasing K_min_” was believed to be closer to the intrinsic fatigue crack resistance at the threshold. However, the “constant K_max_, increasing K_min_” method cannot be used to generate ΔK_th_ values at a specified R ratio and can still introduce loading history effects [14]. The “constant R, increasing K” method was developed to minimize the plastic history effect during fatigue testing, of which the ΔK level starts well below the anticipated ΔK_th_ value and is then raised by a small amount at each step until the crack can grow constantly [14]. Nevertheless, the increment of ΔK at each step needs to be carefully designed because a very fine step is time consuming and a very large step causes reduced accuracy and a divergence of results.

As a common consensus in fatigue threshold tests, most tests have been performed on precracked or notched specimens. Several authors mentioned their elaborate control of the precracking process in order to minimize the introduction of the preliminary plastic zone ahead of the crack tip. Mercer et al. [6], Pippan et al. [7], and several other authors precracked the specimens with far-field compression loading, which is a technique often used for generating precracking in brittle materials. Fatigue precracks introduced in this way are usually very short and produce little influence on the load history [14,15]. However, this method often involves very high compressive stresses (close to the yield stress) and could cause damage in the bulk. Besides the above technique, there have not been many precracking methods used in TiAl alloys. Balsone et al. [9] tried to precrack specimens under tension–tension cyclic loading and kept the K_max_ lower than the initial K_max_ level of the subsequent fatigue testing. Hénaff et al. [4] also applied a tension–tension fatigue loading of R = 0.1 to precrack specimens. However, this precracking technique needs to be carried out very carefully, or it can lead to a premature rupture during the process.

In this paper, the author measured the fatigue thresholds and crack growth resistance curves for a nearly lamellar γ-TiAl alloy (Ti-45Al-2Mn-2Nb-1B at.%) on single-edge notch bending (SENB) specimens and corner-cracked (CC) specimens. The CC specimen is the preferred test piece geometry to characterize the fatigue crack resistance of aeroengine rotors, since the stress state of CC specimens is more similar to that of real turbine attachment, and corner cracks are often found in real turbines [17,18]. Tests were conducted mainly via the load-increasing method under constant R ratios to restrict the loading history effect. A limited number of tests were performed using a load-shedding procedure in SENB specimens for comparison purposes. The influences of notch depth, crack starter morphologies, and precracks on the fatigue threshold were also examined. Rather than concluding which testing method was more superior, the aim of this study was to evaluate the difference in results caused by the various testing methods and the influential factors for lamellar γ-TiAl alloys. It is hoped that this study can provide technical guidance for determining the fatigue thresholds in lamellar γ-TiAl alloys.

## 2. Materials and Experimental Works 

### 2.1. Materials and Specimens

The nominal composition of the γ-TiAl alloy was Ti-45Al-2Mn-2Nb-1B (at.%). This alloy has a nearly lamellar microstructure with a colony size in a range of 40–90 μm. There are only a few colonies of exceptions that are smaller than 40 μm or larger than 90 μm. Equiaxed γ grains with a volume fraction of 11–15% was found among the lamellar colonies. 

**Figure 1:**
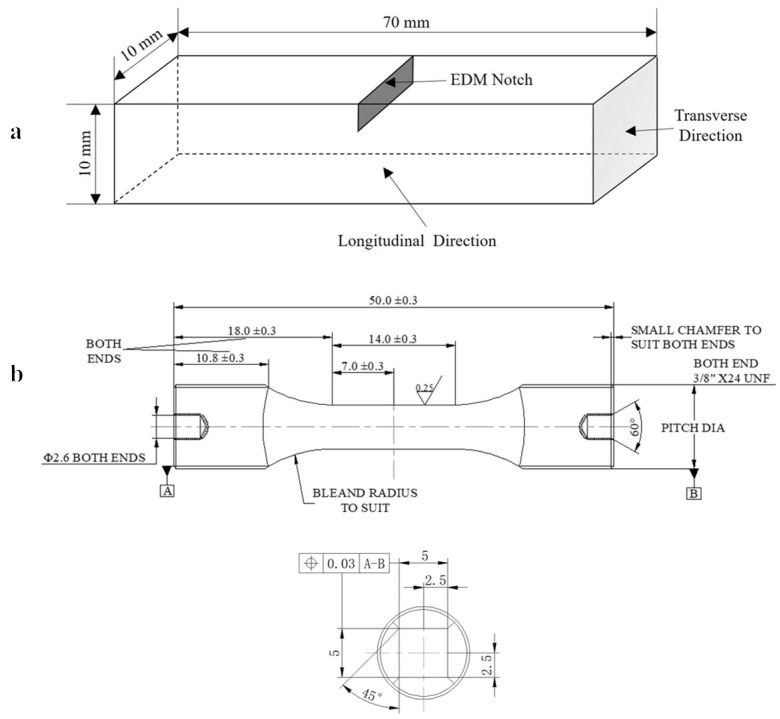
Schematic diagram shows the dimension of the (**a**) single-edge notch bending (SENB) and (**b**) corner-cracked (CC) specimens. The unit of the dimensions is mm.

Single-edged notch bending (SENB) and uniaxial corner-cracked (CC) specimens were machined from the ingots. The dimensions of the SENB and CC specimens are shown in Figure 1. Through-thickness notches in the SENB specimens were about 1.8 mm in depth and 0.25 mm in width. The notches in the CC specimens with different depths (0.2, 0.3, 0.5, and 0.7 mm) were introduced by ultrafine electro-discharge machining (EDM) wires of 30 μm in diameter. The notches were in the middle of the gauge length at one corner of the CC specimens, and the notch front was about 45° to the edge of the square cross-section. Most fatigue tests in the CC specimens were carried out directly from notch roots without precracking. Only two CC specimens were precracked, at room temperature (RT) and 400 °C, for the comparisons.

### 2.2. Fatigue Threshold and Crack Growth Tests

#### 2.2.1. Precracking

As mentioned above, far-field cyclic compressive load is usually employed for precracking brittle materials. The SENB specimens were precracked by far-field uniaxial compressive cyclic loading (R = 0.1, 15 Hz, sinewave) along the longitudinal direction of the specimens using a servohydraulic testing machine (Phoenix F1002, Phoenix Material testing Ltd., UK) with a load cell of 20 kN. A specified peak load of approximately 26 kN was applied, which was high enough to cause plastic deformation ahead of the notch root but not to cause material yielding elsewhere. A localized tensile residual stress field was produced due to plastic yielding at the notch root, which led to limited cracking ahead of the notch. The precrack introduced in this manner was self-arresting and typically about 0.2–0.3 mm. The precrack front was then marked by exposing the sample at 700 ℃ for 30 min. However, the above precrack technique was found not to be applicable for the CC specimens. Therefore, the CC specimens were precracked under tension–tension cyclic loadings of R = 0.1 at room temperature (RT) and 400 ℃ by performing a load-increasing procedure, similar to the load-increase threshold testing procedure described below, and stopped after a sustainable crack growth (~100 μm) was confirmed and detected by the direct current potential drop (DCPD) technique. The subsequent fatigue tests were carried out at 650 ℃ in order to make distinctions from the precracking process. 

#### 2.2.2. Testing Details 

The SENB and CC specimens were tested at RT and 650 ℃ with an R ratio of 0.1 in air. The loading waveform was sinusoidal, and the frequencies used were 15 and 10 Hz for the SENB and CC specimens, respectively. The determination of the fatigue threshold primarily employed a staircase load-increasing procedure with constant load ratios. The initial peak load was selected so that no cracking could be caused over a significant number of cycles, i.e., the resulting ΔK (= K_max_ − K_min_) was below the ΔK_th_ of the material. After confirmation of nongrowth, a small increase of 0.2 kN in the peak load was applied (still maintaining the same load ratio) for another cycle interval (~50,000 cycles), while any crack initiation and growth was monitored. If no crack growth was observed, then another increase in load was applied, and so on. Immediately after a continuous crack growth was observed, the load level was then kept unchanged, and the crack was allowed to grow until a certain crack length was achieved or when the crack growth became unstable. The DCPD technique was utilized throughout the tests to monitor crack growth. The measured lengths of the initial crack (or notch) and the final crack were used to calibrate the potential drop (p.d.) record and obtain crack length data points. The selected load increment (0.2 kN) and the total number of cycles during each load level before the final constant load level (50,000 cycles) ensured a resolution in the ΔK of 0.2 MPa·m^1/2^ and a low growth rate of 1 × 10^−7^ mm/cycle. The tests were stopped when the crack growth rate reached a value more than 10 μV/min in order to mark the final crack length. The final crack length was marked by exposing the specimens at an elevated temperature for about half an hour. The heat-tint temperatures were 500 ℃ and 700 ℃ for the tests carried out at RT and 650 ℃, respectively. Then, all the specimens were opened in air at RT.

The load-shedding (load-decreasing) procedure that was employed followed ASTM standard E647. These tests were conducted on SENB specimens at RT and 650 ℃ under a R ratio of 0.1. The results were compared with those obtained by using the load-increasing method. The initial ΔK level was in the range of 10–15 MPa·m^1/2^, which resulted in an initial crack growth rate of ~1 × 10^−4^ – 1 × 10^−3^ mm/cycle. The load range was then reduced by ~3–10%, once the crack length was about 4 times the size of the cyclic plastic zone for a given ΔK value. This process was repeated until the crack growth rate was less than 1 × 10^−7^ mm/cycle. The corresponding ΔK value was deemed as the fatigue threshold. One test was tested first by the load-increasing method and then by the load-decreasing method (RT and R = 0.1). Another test was conducted with the load-decreasing method first and then followed with the load-increasing method at R = 0.1 and 650 ℃.

The actual initial and final crack lengths were measured from optical images (resolution: 3072 × 2048, magnification: ×50–100) using Axiovision software. The fracture surface observation and microstructural characterization were carried out using scanning electron microscopy (SEM, Jeol 7000F and Jeol 6060)

#### 2.2.3. Data Processing

**Figure 2:**
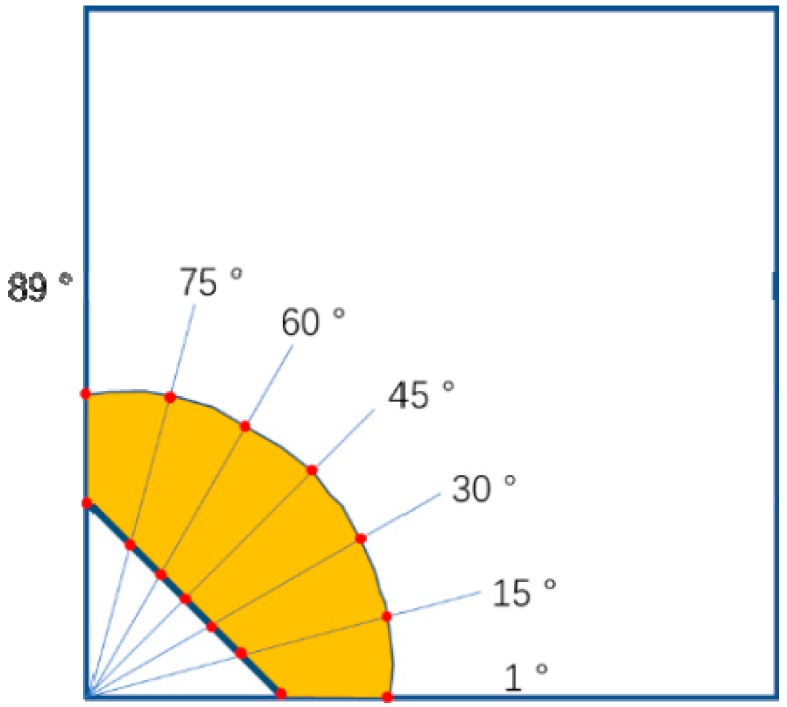
Schematic diagram of the seven-point methodology used for measuring the actual crack length. The red dots indicate the measurement points.

For the SENB specimens, the precrack and final crack lengths were obtained via the nine-point method [19], i.e., an average length of nine equidistantly distributed points at the crack front. In the CC specimens, the initial and final crack lengths were measured by averaging the seven measurements along different angles, as specified in Figure 2. 

## 3. Results

### 3.1. Effect of Loading Schemes—Load-Increasing Method vs. Load-Decreasing Method

All the fatigue testing results are summarized in Table 2. As can be seen, the fatigue threshold value more or less altered with the different factors, such as loading methods, specimen configurations, notch depth, and crack starters. The most significant variations of the ΔK_th_ values were found between the tests conducted by different loading procedures. As the load-decreasing method usually requires a certain amount of crack extension, the cross-section of the CC specimens was limited, therefore, it was difficult to carry out the load-decreasing method on the CC specimens. As a result, the load-increasing method and load-decreasing method were only compared on the SENB specimens.

**Figure 3:**
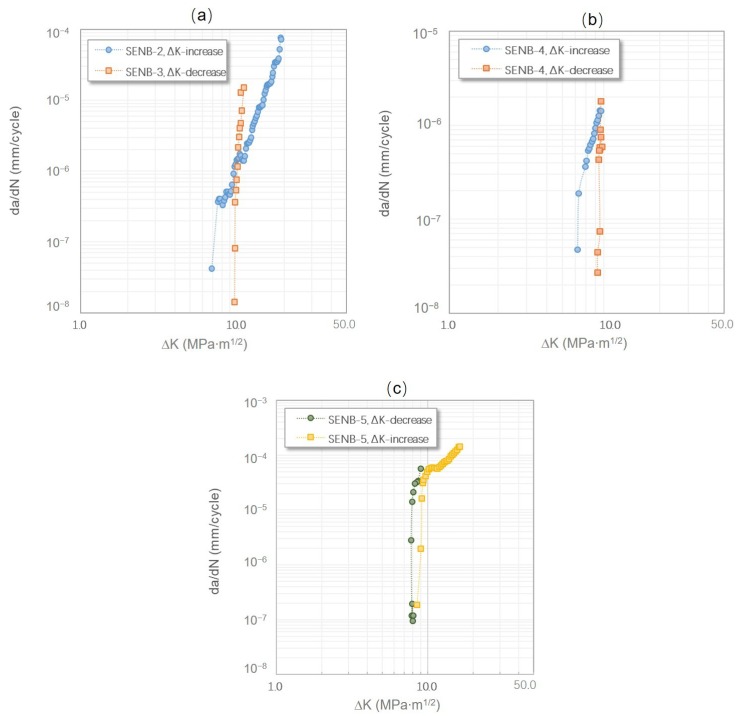
FCG curves of SENB specimens tested with different loading methods: (**a**) Specimens SENB-2 and SENB-3 were tested via solo load-increasing method and load-decreasing method, respectively, (**b**) Specimen SENB-4 was tested first by the load-increasing method and then by the load-decreasing method, and (**c**) Specimen SENB-5 was tested first by the load-decreasing method and then by the load-increasing method.

Clear differences, as shown in Figure 3a, were also observed between the FCG curves generated via these two loading schemes. The FCG curve of the load-increasing method showed gradual increases in the FCG rate with increasing ΔK values, while the FCG curves of the tests obtained by the load-decreasing method were nearly a vertical line, ending up with relatively higher ΔK_th_ values and lower FCG rates. As shown in Figure 3b, the FCG curve obtained through the load-increasing then decreasing method exhibited a similar trend to the above observations. However, if the specimen was tested through the load-decreasing then increasing method, the ΔK_th_ values and FCG rates obtained from the former method were lower than those of the latter method (as can be seen in Table 2 and Figure 3c). 

In summary, if only one loading method was used throughout the tests, the load-increasing method resulted in lower ΔK_th_ values but higher FCG rates. If more than two loading methods were applied to a test, no matter what loading method is applied first, the latter loading method always resulted in higher ΔK_th_ values, whereas the FCG rates were normally slightly lower at the threshold if they were obtained from the load-decreasing method, regardless of the loading method sequence.

### 3.2. Effect of Specimen Notch Depth and Configuration on Fatigue Threshold

**Figure 4:**
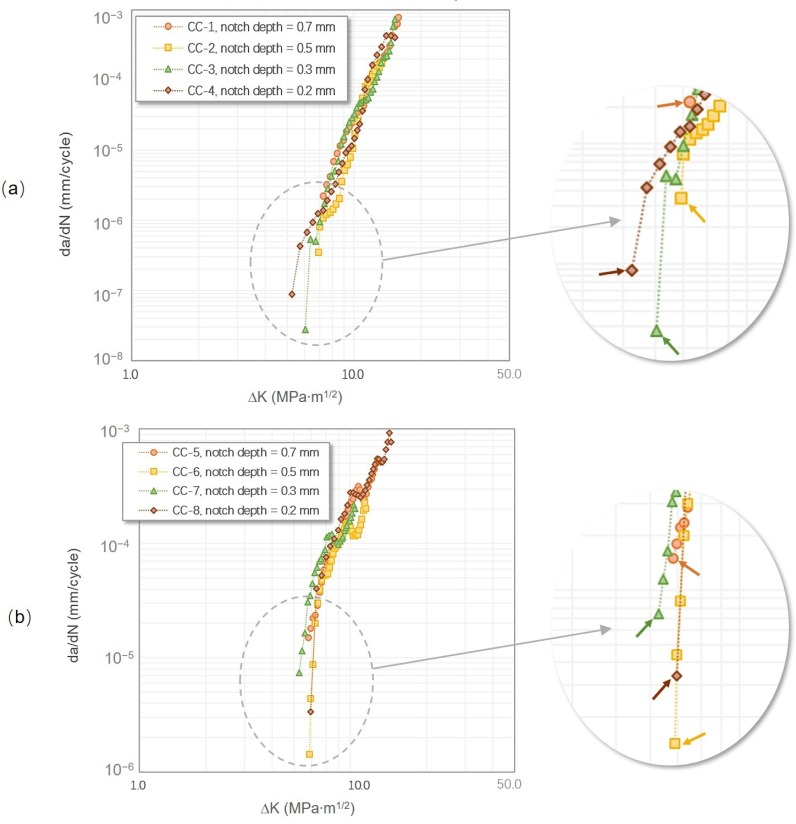
FCG curves of tests carried out using the ΔK-increasing method in the CC specimens: (**a**) at room temperature (RT) with an R ratio of 0.1 and (**b**) at 650 °C with an R ratio of 0.1. For comparison purposes, all the tests were directly started from the notch root without precracking. The fatigue threshold values are indicated by arrows.

As can be seen in Figure 4, the results of the CC specimens coincide well with each other at both testing temperatures, showing only subtle differences of the near-threshold behaviors. Regardless of the specimen configuration, the FCG curves obtained at RT were steeper than those obtained at 650 °C and show no clear fatigue regimes. This observation was consistent with the low-ductility nature of the γ-TiAl alloys at RT and indicates a significant increase of ductility at the elevated temperature. Besides, at RT, before the ΔK_th_ value was reached, a small and limited amount of crack growth could be found at two or three loading steps earlier, while it was not seen at 650 °C. Therefore, the ΔK_th_ value measured via the load-increasing method in this study is defined as the minimum ΔK level that can ensure a continuous crack growth from the start to the final failure.

It can be seen in Table 2, at RT, that the ΔK_th_ values increased with an increasing notch depth, whereas the peak load (P_max_) at the threshold was on the contrary. In addition, for the fatigue threshold regime, the FCG rates of the short-notch tests (0.2 and 0.3 mm) were relatively lower at the beginning, increasing dramatically once the crack growth was triggered, and finally returning to a gradual trend. Although the starting FCG rates of the long-notch tests (0.5 and 0.7 mm) were relatively higher, no sudden changes were found as in the short-notch tests. However, such trends on the P_max, th_ (the maximum load at threshold) and ΔK_th_ values were not seen at 650 °C, i.e., they were independent of the notch depth. Interestingly, at both testing temperatures, the highest starting FCG rates were both found in the specimens with the longest notches of 0.7 mm, and there was always an obvious “jump” of the initial FCG rates for the specimens with the shortest notches of 0.2 mm. 

**Figure 5:**
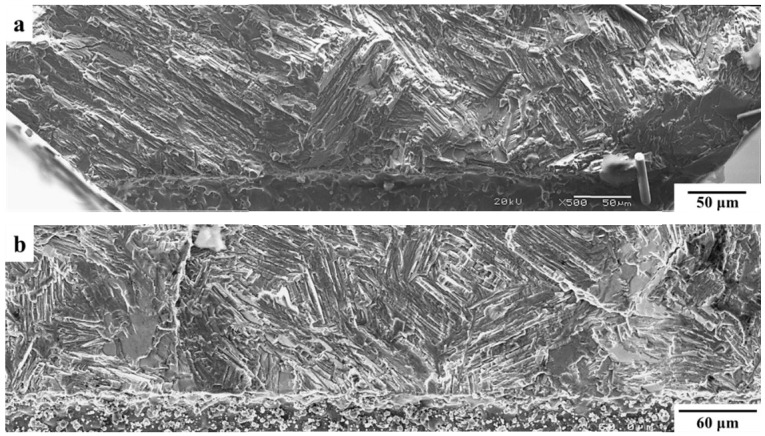
Fatigue crack initiation areas of (**a**) Specimen CC-4 and (**b**) Specimen CC-1, which were both tested at RT with an R ratio of 0.1. The notch depths of CC-4 and CC-1 were 0.2 mm and 0.7 mm, respectively.

**Figure 6:**
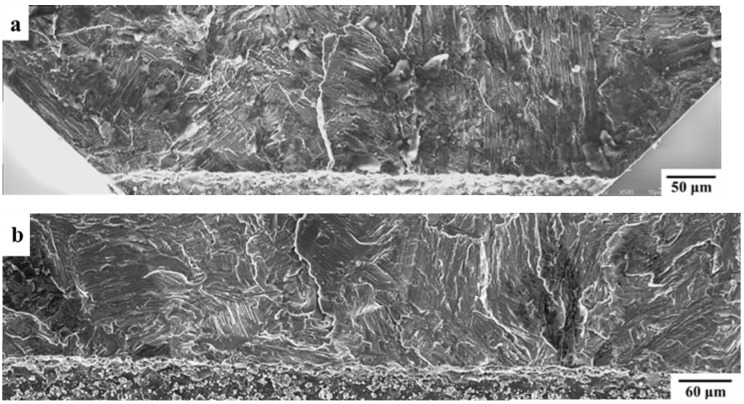
Fatigue crack initiation areas of (**a**) Specimen CC-8 and (**b**) Specimen CC-5, which were both tested at 650 °C with an R ratio of 0.1. The notch depths of CC-5 and CC-8 were 0.7 mm and 0.2 mm, respectively.

In Figure 5 and Figure 6, the fatigue crack initiation areas of the specimens with the shortest and longest notches, i.e., the fracture surfaces of the two most distinctive results, are compared for the tests carried out at RT and 650 °C. As shown in Figure 5, generally, the fracture surface of Specimen CC-4 (with the shortest notch) was relatively rough, showing that a prominent colony failed at a high angle. The fatigue crack initiation area of the specimen with the longest notch (CC-1) was fairly flat with several interlamellar facets, which were perpendicular to the loading axis. Regarding the fatigue crack initiation areas of the specimens tested at 650 °C, as shown in Figure 6, there was no significant difference between the specimens with the shortest and longest notches.

### 3.3. Tests Started from Precrack vs. from Notch

**Figure 7:**
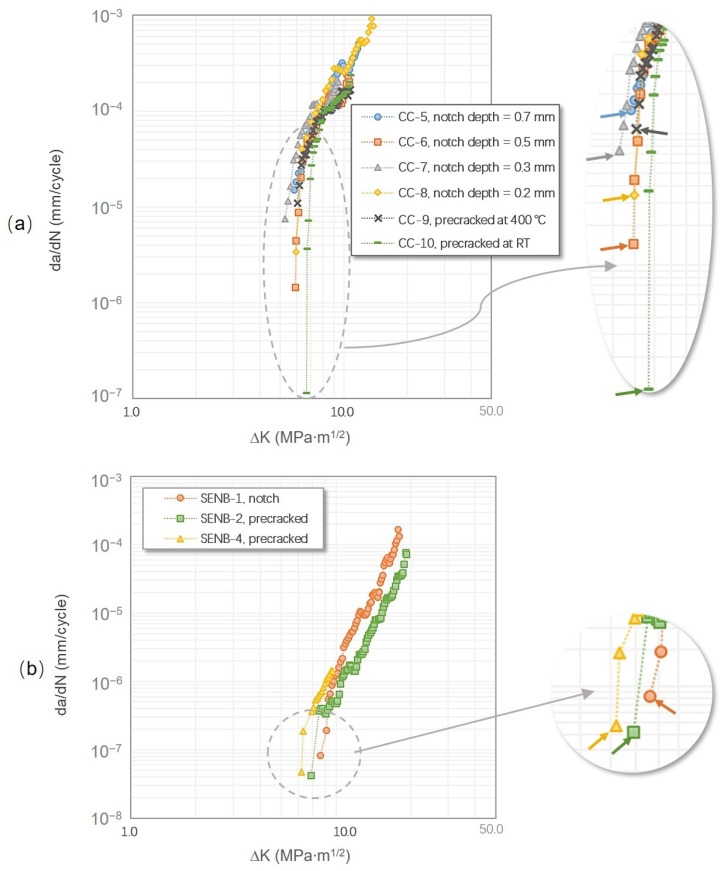
Comparison of the FCG curves of specimens with and without precrack. All the tests were carried out using the load-increasing method: (**a**) the test conducted in the CC specimens at 650 °C and R = 0.1, of which two specimens were precracked at RT and 400 °C and then tested, and (**b**) the tests conducted in the SENB specimens at RT and R = 0.1.

The FCG curves of the tests that started from the notches and precracks are compared in Figure 7. The nearly consistent FCG curves in Figure 4 and Figure 7 indicate the repeatability of the results obtained by employing the load-increasing method. Due to the several limitations mentioned in the experimental details, the precracked CC specimens were only tested at 650 °C with an R ratio of 0.1. The results were compared with the tests directly started from the notches, as shown in Figure 7a. It can be seen that the precracks introduced at different temperatures had different influences on the fatigue threshold of the tested alloy. The near-threshold properties of the specimen precracked at 400 °C was similar to the results of the CC specimen with a notch depth of 0.7 mm, of which the near-threshold FCG rate was relatively higher. In contrast, the specimen precracked at RT not only exhibited the highest ΔK_th_ value but also the lowest FCG rate at the near-threshold regime. 

**Figure 8:**
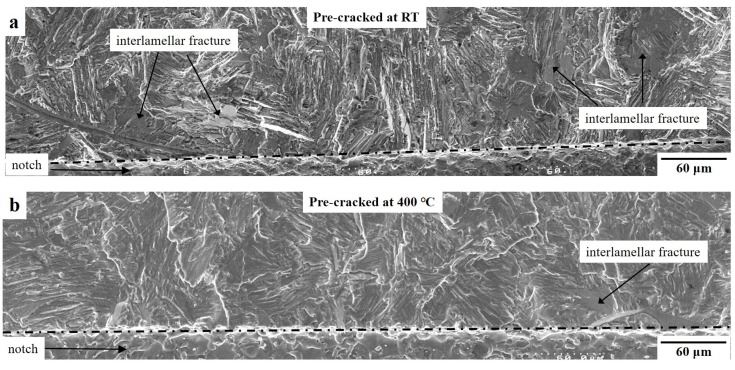
Precracks generated by cyclic tension–tension loading with an R ratio of 0.1 at (**a**) RT and (**b**) 400 °C.

By examining the fractographic morphology of these two different types of precracks, the distinctions between them are revealed in Figure 8. The precrack generated at RT was fairly rough, containing several large interlamellar fractures and uneven translamellar fractures. In contrast, the fracture surface generated at 400 °C was relatively smoother, and the dominated fracture mode was a translamellar fracture.

**Figure 9:**
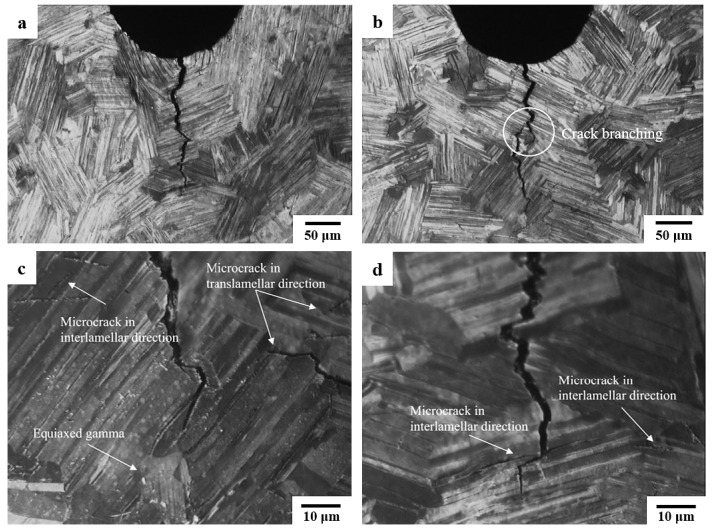
Optical images show a precrack in the SENB specimen generated by far-field cyclic compression load and heat tinted at 700 °C: (**a**,**b**) show the two side surfaces containing the precrack, and (**c**,**d**) show the details of microcracks found along the precrack.

In order to investigate the differences between the precracked and the notched specimens at RT, the tests were carried out in SENB specimens with an R ratio of 0.1. The test that began from a notch root showed the highest ΔK_th_ value and FCG rate at the threshold compared to the two specimens with precracks. It is noteworthy that the P_max_ at the threshold (in Table 2) of the notched specimen was about 0.6–0.8 kN higher the those of the precracked specimens, indicating an increased difficulty for starting a crack from a rounded notch root than from a sharp precrack tip. An example of a precrack introduced by far-field cyclic compression load is shown in Figure 9. A strong contrast of the sharpness of a notch and a precrack is revealed. The precrack propagated mainly via the translamellar fracture mode and a small fracture of the interlamellar can be seen. Microcracks were also observed along the precrack either in translamellar or interlamellar directions. 

## 4. Discussion

### 4.1. Fatigue Threshold in Lamellar γ-TiAl alloys

In the absence of loading history and precracks, i.e., tests were conducted using the load-increasing method and started from a notch root, the fatigue behaviors at the threshold were expected to be similar to that of the “constant K_max_, increasing K_min_” method, of which the ΔK_th_ values are interpreted as the intrinsic fatigue crack growth resistance since the crack closure is considered to be an extrinsic property of the materials [13]. This intrinsic fatigue threshold is supposed to be only affected by the plastic zone and microstructure ahead of the crack tip (notch root), external force, and environment. 

In the fatigue crack threshold regime, where the plastic zone size is normally smaller than the microstructure units, crack initiation is highly sensitive to the microstructure of the materials and applied K_max_. As observed in a previous in-house study, fatigue cracks in lamellar TiAl alloys prematurely formed at the lamellar interface causing a “short crack” behavior [20] and a so-called “crack-arrest” phenomenon [21]. This can explain the earlier small crack growth observed at load levels below the fatigue thresholds at RT. When the ΔK level was high enough to trigger a continuous crack growth, in general, cracks started to grow translamellarly. The microstructure influence is often observed at low R ratios as a result of crack closure effect, which can retard the crack initiation and growth. The lamellar structure exhibits superior FCG resistance than the other microstructures of the TiAl alloy, because most of the crack closure effects, e.g., plasticity-induced crack closure, roughness-induced crack closure, crack deflection, and crack-bridging effect, can be more frequently found during the crack propagation in the lamellar structure than in other microstructures [3,22]. However, the microstructure relevant crack closure effects can be reduced or even eliminated when the stress ratio is high enough to give K_max_ values that are greater than crack closure stress intensity (K_cl_), i.e., K_eff_ = K_max_ – K_cl_ > 0 [22]. 

The “jump” in the initial FCG rates found in the specimens with short notches at RT was probably associated with the relatively high peak load levels (shown in Table 2). The highest P_max,th_ value of CC-4 with the shortest notch was 1.6 kN higher than that of CC-1 with the longest notch. Such a high stress level was able to cause an instantaneous, large-scale delamination in colonies at various orientations and thus led to a dramatic change in the crack length and FCG rate. Indirect evidence can be seen in Figure 5 that shows that the fracture surface generated at the higher peak load was similar to that caused by monotonic loadings. 

In addition, for the tests at RT and low R ratio, an inference can be proposed that the dispersion of the ΔK_th_ values was probably increased due to the short-notch fronts. As the fatigue threshold behavior under such a condition is highly sensitive to the localized microstructure along or near the notch root, and the fewer the colonies along a notch root, the extremely oriented colonies, i.e., at the hardest orientations and the softest orientations are more dominant. For example, if there are only 5–6 colonies ahead of the notch front, one large colony at the soft orientations (perpendicular or 45° to the loading axis) can result in a significant reduction of the ΔK_th_ value, while colonies at hard orientations (parallel to the loading axis) can effectively retard the crack initiation. 

By combining the results in Table 2 and Figure 6, why the effects of notch depth were not obvious at 650 °C can be explained. Although the difference of the P_max,th_ values (ΔP_max,th_ = 3.6 kN) between the specimens with the shortest and longest notches was prominent, their fatigue crack initiation areas were very similar to each other, indicating that there are more predominant fracture mechanisms at 650 °C than the external forces. Gloanec [23] studied the deformation behavior on a cast Ti-48Al-2Cr-2Nb alloy with a near lamellar structure at 750 °C. It was found that long and pointed dislocations were the primary deformation morphologies at this temperature, indicating the possible increased activity of cross slip and climb. The effects of the microstructure in the fatigue threshold regime is likely to be reduced by the reason that microstructural barriers, including lamellar and colony boundaries, can be overcome more easily as a result of the growing diffusion activity of dislocation at an elevated temperature. 

One factor that has not been mentioned in the results section is the effect of specimen configuration. The parameters of notches, such as notch root radius, notch depth/width, and notch shape, in the CC specimens and SENB specimens were considerably different, which can lead to variations other than individual configuration effects. Additionally, the SENB specimens had much longer crack fronts (~10 mm) than the CC specimens (≤0.8 mm), i.e., there were more colonies ahead of the crack front in the SENB specimens, and thus they were less sensitive to large colonies at the weak or hard orientations at the thresholds. The specimen configuration can also be influential on the fatigue results. For example, works by other authors on CT specimens and CC specimens showed that fatigue crack growth rates of CT specimens were higher than that of CC specimens by a factor of 2 under the same testing condition [17,24]. An explanation, based on finite element modeling given by Zhao et al. [24], is that the T-stresses in the CC specimens and CT specimens are negative and positive, respectively. Meanwhile, a negative T-stress can stabilize the crack growth in the Mode I direction. A similar method can also be applied to investigate the above issues between the CC and the SENB specimens. 

### 4.2. Effect of Loading Schemes

It is apparent that sole loading schemes, the load-increasing method and the load-decreasing method, result in dramatic distinctions of fatigue behaviors, as described in Section 3.1 and Figure 3. Such differences can be directly correlated with the most logical variation between the two loading methods—length of the crack wake. The crack closure concept has been widely used to explain the crack retardation phenomena in lamellar γ-TiAl alloys. Most crack closure mechanisms in lamellar TiAl alloys are relevant to the build-up of the crack wake during cycling. As mentioned above, these crack wake relevant crack closure behaviors can be a constrained crack tip introduced by the plastic zone because of the previous loading history, a subdued crack opening due to the hold-up by unbroken lamellae at “hard” orientations, localized stress offset due to the mismatch and contact of crack flanks during a closing-to-opening cycle, or crack flank adhesion due to oxides at elevated temperatures [4,7,9,20,21,22]. 

**Figure 10:**
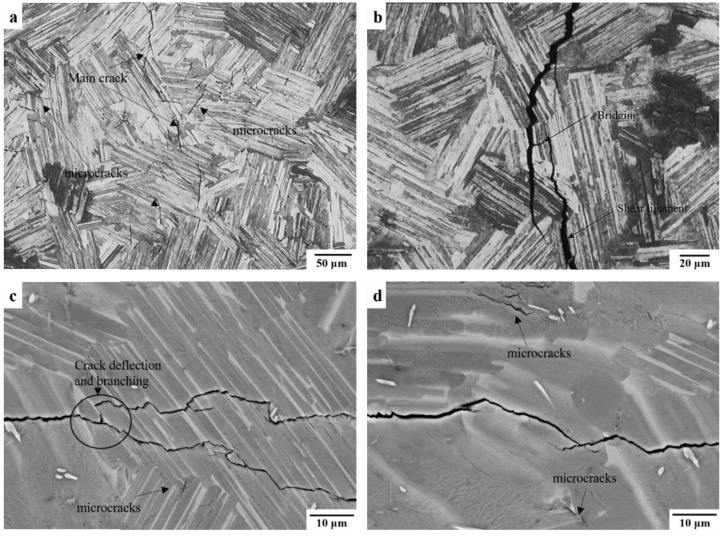
Toughening mechanisms were found in (**a**,**b**) SENB specimens and (**c**,**d**) CC specimens, including microcrack shielding, bridging by shear ligaments, crack deflection, and branching. Both specimens were tested at RT with an R ratio of 0.1.

Along with these extrinsic crack closure behaviors, intrinsic toughening mechanisms, including crack tip deflection and microcracking shielding at RT, as well as crack tip blunting [21,22] at an elevated temperature, can be also induced even under a short period of fatigue loading, further reducing the driving force for FCG ahead of the crack tip. Some of these intrinsic toughening behaviors have also been found in this study, examples are given in Figure 10. Although the intrinsic toughening behaviors should be considered as part of the intrinsic resistance against crack initiation and growth, they are hardly extracted from the total contribution of the total crack closure effect, affecting the evaluation of the ΔK_th_ values and FCG rate if a loading history is inevitable. 

**Figure 11:**
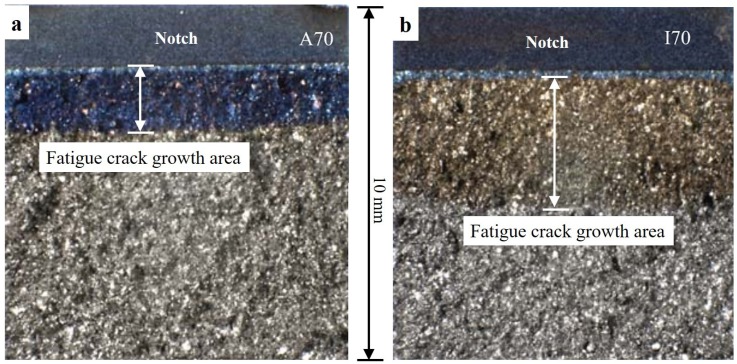
SENB specimens tested with different loading methods at RT with an R ratio of 0.1: (**a**) A70 tested using the load-decreasing method and (**b**) I70 tested using the load-increasing method. The fatigue crack growth regions in both specimens are indicated by the white arrows. The remaining untinted areas are fracture areas.

Although the ASTM standard E647, also known as the load-shedding/decreasing method, and the “constant K_max_, increasing K_min_” method are the most common loading schemes for measuring materials’ ΔK_th_ values, they both inevitably introduce loading history. By using the load-decreasing method, before a crack stops growing, a certain length of crack wake and large plastic strains are produced at high loads. This plastic loading history leads to a significant crack closure effect, and thus closing the crack opening prematurely [25]. As shown in Figure 3 and Figure 11, the crack propagation under the load-decreasing method is normally less than using the load-increasing method, and thus has fewer data points and a steeper FCG curve. Besides, the up-end of the FCG curves obtained from the load-decreasing method cannot represent the trend of fracture toughness, since the starting stress is specified rather than an infinite approaching value, as compared to using the load-increasing method. The “constant K_max_, increasing K_min_” method is considered as unaffected by the plasticity-induced crack closure, of which the ΔK_th_ values are regarded as a pure intrinsic material fatigue crack resistance. However, the procedure of this method makes it hard to determine the FCG behaviors at a specified R ratio. In addition, the K_max_ value needs to be carefully selected, because it has been proved to have a marked influence on threshold results and may cause creep effects especially at high temperatures [26,27]. An insignificant amount of loading history effect still exists, because the ΔK_th_ value is also determined by closing a crack, which is similar to the “constant R, decreasing K” method. Therefore, the crack closure is still influential on the K_op_/K_cl_ values (K_op_ and K_cl_ are the stress intensity factors for the open and closed crack, respectively), as long as there are crack opening–closing cycles [22].

**Figure 12:**
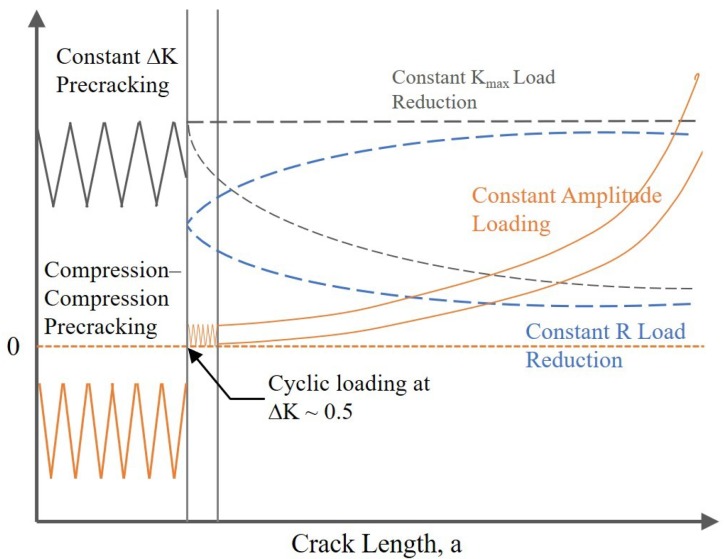
Fatigue threshold test methods compared in the work of Forth et al. [25]: (1) constant K_max_ load reduction, (2) constant R load reduction, and (3) constant amplitude loading.

Other authors [8,14,28,29,30] have also utilized a “constant amplitude (CA) load” method to determine the fatigue crack threshold values that is believed to be the other loading method to minimize or eliminate load history effects. A schematic diagram of the above fatigue threshold methods is shown in Figure 12. To achieve a “history free” state, the specimens are normally cyclic precracked under a fatigue compression loading and are then allowed to grow out of the tensile residual stress induced in the plastic zone under a constant amplitude loading. The CA loading method is carried out with a constant amplitude and a specific R value. To a certain extent, the CA method and the load-increasing method in this study can be regarded as identical for measuring the fatigue threshold as well as in the precracking procedures. For both testing methods, “trial-and-error” procedures were applied to evaluate the initial fatigue loading for materials of unknown threshold values, i.e., the tests were started with a load level below the anticipated ΔK_th_, if no appreciable crack growth was observed, then higher loads were applied until steady crack growth occurred. 

For some alloys that failed in a ductile manner, i.e., with a flat and straight crack surface, such as AA7075-T7351 and 4340 steel, the testing methods did not influence the fatigue threshold and near-threshold behaviors significantly; however, microstructure sensitive alloys that failed with rough fracture surfaces, such as γ-TiAl alloys, Ti-6Al-4V, AA7075-T7351, and Inconel-718, were easily affected by the loading procedures [30]. Moreover, even a load-increasing test method can generate higher ΔK_th_ values and a lower near-threshold FCG rate if the loading history pre-exists, as shown in Figure 3c. It is worth considering whether crack starters (precrack and notch) and the methods of introducing them can have an effect on the fatigue threshold behaviors of lamellar γ-TiAl alloys, and if so, how to minimize the effects.

### 4.3. Effect of Fatigue Crack Starting State

The effects of fatigue crack starter on fatigue threshold measurement can be related to plastic zone and stress concentration factors ahead of the crack tip as well as the morphology of a crack starter. 

#### 4.3.1. Compression–Compression Precracking Method

After realizing that the traditional load-shedding method can introduce an unexpected loading history and lead to higher fatigue threshold results, “history free” testing methods, mentioned above, have become increasingly used to measure fatigue thresholds, especially for microstructural sensitive materials and at low R ratios. Most of these “history free” test methods normally start crack growth from sharp precracks, which are regularly induced by a “compression–compression” precracking method in order to minimize the appreciable load history effects. Nevertheless, if compression overloads happen during the precracking, the ΔK_th_ value for the R-curve and short fatigue crack behaviors of stepwise load-increasing amplitude tests can be affected because of the antishielding induced by the plastic deformation, as tested by Zhou et al. [31] both experimentally and numerically. 

**Figure 13:**
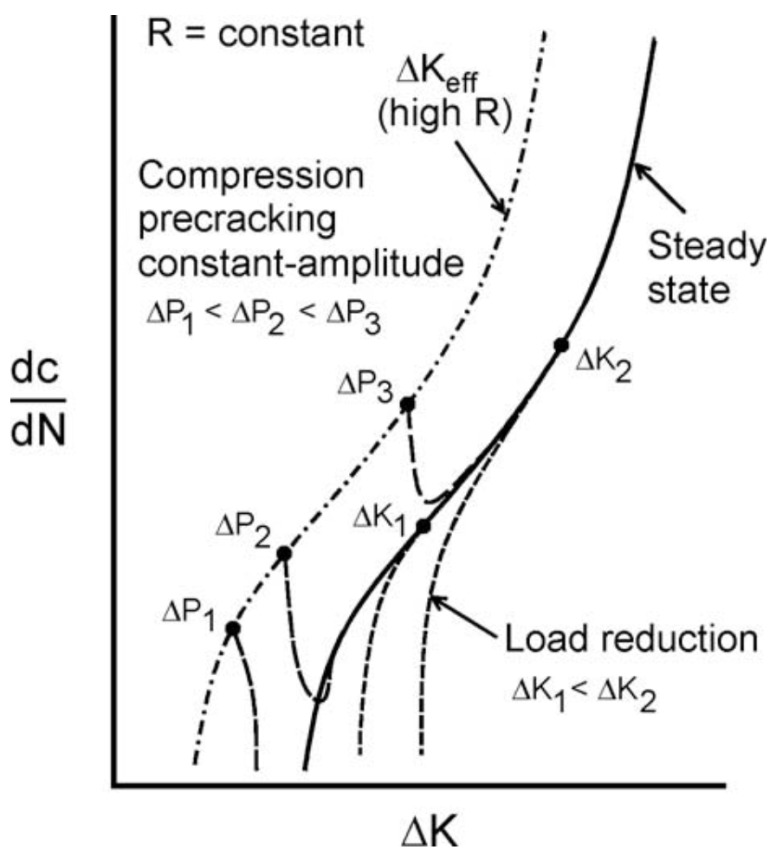
Typical FCG curves generated by the compression precrack constant amplitude method and load-shedding method [30].

Moreover, for a compression precracking constant amplitude (CPCA) fatigue threshold test, tensile residual stresses (induced by compressive yielding at the notch during precracking) can result in a compressive plastic zone, which leads to the “crack-arrest” phenomenon and a rapid drop of FCG rate in the beginning if the initial tensile loading is below that required for the ΔK_th_ value (as shown in Figure 13). The steady state can be achieved only if the crack propagates several times longer than the compressive plastic zone [30]. As mentioned above, the “crack-arrest” phenomenon was also observed in this study at RT both in tests started from precracks and notches, whereas such a “valley” of near-threshold FCG rate in the CPCA tests was not seen. For the SENB specimens tested in this study, which were also precracked by compressive cyclic loads, it is not possible to fully explain the absence of such a reduction of the FCG rate at the threshold regime due to scarcely reported precracking details. The reasons were speculated as follows: (1) there was a compressive plastic zone ahead of the notch that was not comparable to the initial fatigue loads, (2) localized residual stresses may be dissipated because of microcracks around the precracks, normally found in brittle materials [32] (as shown in Figure 9), or (3) the brittle nature of γ-TiAl alloys limits plastic formation, thus fewer constraints were in the plastic zones.

#### 4.3.2. Precrack vs. Notch

Normally, precracking is a necessary procedure for fatigue tests to introduce a sharp crack initiator and reduce the initial stress level for starting crack growth. However, in some circumstances, as for the CC specimens used in this study, where the common precracking approaches are not applicable, starting a fatigue test directly from a notch could be risky because of high stresses inducing material yielding. Although it has been proved that fatigue cracks in this lamellar alloy can be initiated directly from a notch root under stress levels well below its yield stress at temperatures from low to high, it should be kept in mind that the radius of the notch root in CC specimens was very small (~30 μm). 

Despite an average ΔK_th_ value of 5.7 MPa·m^1/2^ of notched specimens, this was slightly lower than the results of precracked specimens at 650 °C. The results of the notched specimens are considered as more representative for the intrinsic crack resistance of the alloy because nearly no loading history or environmental influence were involved to alter the subsequent tests. Nonetheless, Trail and Bowen [33] manifested the notch strengthening effect in a lamellar Ti-48Al-2Mn-2Nb alloy that increases with an increasing stress concentration factor, K_t_, of notches. Hence, further work should consider how much notch geometry would affect the fatigue threshold in the lamellar γ-TiAl alloys. 

It is not surprising that the results of the notched and precracked SENB specimens were converse to the trends of the CC specimens. The tension–tension cyclic load and compression–compression cyclic load can result in two adverse residual stress fields—namely, compressive residual stress and tensile residual stress, respectively. If as previously conjectured, the plastic zone generated after precracking can be dissipated or diminished in this lamellar microstructure as a result of intrinsic retardation mechanisms, the residual stresses at the notch root can possibly play roles in promoting or retarding the fatigue crack initiation. 

Apart from heat exposure effects, the morphology of precrack flanks is suspected to have significant effects on the fatigue threshold behaviors in the Ti4522XD alloy, as indicated by the obvious distinctions between the FCG curves (Figure 7a) and fracture surfaces (Figure 8) of specimens precracked at RT and 400 °C. The rougher fracture surface of the precrack generated at RT can result in several crack closure effects to hold back the crack growth so that a higher ΔK_th_ value and a much lower FCG rate can be achieved.

### 4.4. Additional Considerations

Two additional considerations for the stepwise load-increasing method—the scale of loading steps and the length of load holding intervals—are also worth mentioning. 

**Figure 14:**
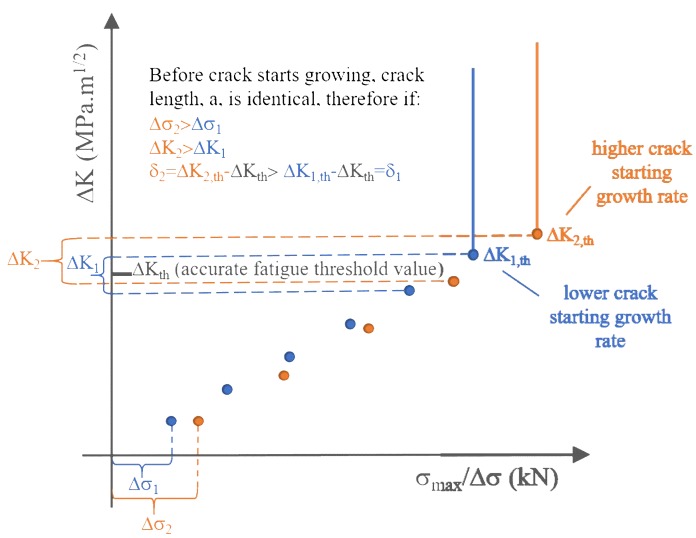
Effect of stress incremental interval on stepwise load-increasing method.

As the accurate ΔK_th_ value could lie in any value within the incremental interval of ΔK, a large increment at each step is thus likely to cause dispersions of the ΔK_th_ values and initial FCG rate, as schematically illustrated in Figure 14. Therefore, careful attention is needed when referring to small distinctions of different fatigue threshold results using an increasing load method, as such inapparent variations may be caused by the less conservative loading increments rather than the discrepancy of the inherent fatigue properties of materials. The increment of each loading step for both testing configurations was 0.2 kN, which corresponds to ~0.5 MPa·m^1/2^ for the SENB specimens and ~0.13–0.25 MPa·m^1/2^ for the CC specimens with respect to the notch depths altering from 0.2 to 0.7 mm. These increments are believed reasonable since even the results of the SENB specimens tested with a larger ΔK increment showed insignificant variations in the fatigue threshold regime.

Another concern with the load holding intervals was the “crack-arrest” phenomenon. According to the literature [2,21,34], different from the above-mentioned crack arrest caused by the compressive plastic zone of precracks, the crack arrest observed in lamellar γ-TiAl alloys is normally caused by microstructural barriers, such as grain boundaries or colony interface. We investigated the question of whether the load holding interval was long enough to ensure that there was no further crack growth after a longer holding period, especially after the crack-arrest phenomenon appeared. In this study, the load holding intervals for the SENB specimens and CC specimens were 1 h and 2 h, respectively. To answer the question, several specimens were left at loading steps where a limited crack extension was observed for more than 12 h (>4.3 × 10^4^ cycles), and no further crack growth was observed. This implies that the crack-arrest phenomenon in lamellar γ-TiAl alloys is energy predominant (or stress predominant) rather than a time-dependent behavior.

Moreover, with the development of microscale and nanoscale measurement techniques, such as microcantilever and microsplitting [35,36], the localized residual stresses and toughness of single phases can be quantified. It is hoped that the fatigue threshold in the macroscale can be interpreted as an integral result of mechanical properties of each microstructural unit ahead of the crack tip. 

## 5. Conclusions

This study investigated and discussed the effects of influential factors, such as loading schemes, geometry of crack starters, precracking approaches, and specimen configurations, on the fatigue crack growth threshold and near-threshold behaviors in a lamellar γ-TiAl alloy at both low and elevated temperatures. As a microstructural sensitive material, the fatigue threshold behaviors of a lamellar TiAl alloy can be affected by the above factors, which can be manifested either by the results in this study or by other authors. Such effects are mainly associated with the crack closure mechanisms, such as crack bridging, microcrack shielding, crack deflection, surface contact retardation, and other crack closure mechanisms, often found in lamellar structures. Several conclusions can be drawn upon the observations and discussions.

(1) The load-decreasing method results in higher ΔK_th_ values and steeper fatigue crack growth curves than the load-increasing method. However, a preceding loading history can also lead to higher ΔK_th_ values even for load-increasing tests;
(2) Notch depths can alter the ΔK_th_ values due to significant variations of the starting load levels, especially at RT and low R ratios, where the microstructure plays a dominant role on fatigue crack initiation;
(3) Precracking is not necessary for fatigue tests in lamellar TiAl specimens if the notch front is very sharp. The tests from precracks and notches show a perceptible difference at the fatigue threshold regime. However, the results from specimens without precracking are believed to offer a more accurate intrinsic crack resistance of the alloy;
(4) Temperature has a significant influence on both the fatigue crack threshold and crack growth behaviors in this lamellar Ti4522XD alloy. In general, the FCG curves are relatively straight at RT, whereas they are more similar to those of conventional ductile materials at high temperatures, indicating increased ductility. At RT, the microstructure plays a more predominant role in the fatigue crack threshold regime and thus leads to an obvious divergence due to microstructural variation, especially for the notches with a short front. As the temperature increases, the thermal driving force becomes more superior to the microstructural effects, resulting in less scattered ΔK_th_ values no matter how much the influential factors vary.

In the damage tolerance design approach, fatigue crack growth threshold values are commonly considered to be a criterion below which the component is free of fatigue damage. Hence, the accurate interpretation and measurement of the materials’ fatigue thresholds and near-threshold behaviors are crucial for their implementation. If the test procedure involves factors other than the inherent fatigue properties of the material, switching the criteria to higher boundaries, the unconservative results will lower the safety class of the materials. 

## Figures and Tables

**Table 1:** Examples of specimen conditions and testing methods on measuring the fatigue threshold values (ΔK_th_) and fatigue crack growth (FCG) curves in lamellar TiAl alloys.

Authors	Material	Specimen Configuration	Load Case	Testing Method
Gnanamoorthy et al. [3]	Ti-46Al (at.%) + Ti-47Al-3Nb (at.%)	rectangular bar	4-point bending	K-decreasing method
Hénaff et al. [4]	Ti-48Al-2Mn-2Nb (at.%)	compact tension	tension–tension	/
Chan and Shih [5]	Ti-47Al-2Cr-2Nb-0.2B (at.%)	compact tension	tension–tension	prescribed ΔK level + higher ΔK level
		rectangular bar	3-point bending	/
Mercer et al. [6]	Ti-48Al-2Cr-2Nb (at.%)	rectangular bar	3-point bending	ΔK-increasing method
Pippan et al. [7]	Ti-46.5Al-4(Cr, Nb, Ta, B) (at.%)	rectangular bar	bending	ΔK-increasing method
Hamada et al. [8]	Ti-48Al (at.%)	compact tension (cylindrical)	tension–tension	constant load amplitude
Balsone et al. [9]	Ti-46.5Al-3Nb-2Cr-0.2W (at.%)	compact tension	tension–tension	ΔK-decreasing /constant load amplitude
Campbell et al. [10]	Ti-47.7Al-2Nb-0.8Mn, Ti-47Al-2Nb-2Cr-0.2B, Ti-47Al-2Cr-2Nb, Ti-47.3Al-2.3Nb-1.5Cr-0.4V (at.%)	compact tension	tension–tension	Variable ΔK/constant R/load-shedding (ASTM standard E647)
Gloanec et al. [11]	Ti-48Al-2Cr-2Nb (at.%)	compact tension	tension–tension	Constant load ratio/constant K_max_
McKelvey et al. [12]	Ti-47.4Al-1.9Nb-0.9Nb (at.%) – 1 TiB_2_ (vol.%)	disk-shaped compact tension	tension–tension	Load-shedding

**Table 2:** Summary of fatigue threshold and crack growth tests in SENB and CC specimens, including fatigue crack starter, loading methods, maximum load at threshold, and fatigue threshold values.

Specimen ID	Testing condition		Crack Starter	Notch depth (mm)	Loading method	P_max_ at threshold (kN)	ΔK_th_ (MPa·m^1/2^)
	T (°C)	R					
**CC-1**	RT	0.1	notch	0.7	load-increase	5.6	7.2
**CC-2**	RT	0.1	notch	0.5	load-increase	5.8	7.0
**CC-3**	RT	0.1	notch	0.3	load-increase	7.0	6.2
**CC-4**	RT	0.1	notch	0.2	load-increase	7.2	5.2
**CC-5**	650	0.1	notch	0.7	load-increase	4.6	5.4
**CC-6**	650	0.1	notch	0.5	load-increase	5.5	6.0
**CC-7**	650	0.1	notch	0.3	load-increase	6.2	5.3
**CC-8**	650	0.1	notch	0.2	load-increase	8.2	5.9
**CC-9**	650	0.1	precracked (400 °C)	0.5	load-increase	4.4	6.0
**CC-10**	650	0.1	precracked (RT)	0.5	load-increase	4.8	6.7
**SENB-1**	RT	0.1	notch	1.8	load-increase	3.4	7.6
**SENB-2**	RT	0.1	precrack	1.8	load-increase	2.8	6.9
**SENB-3**	RT	0.1	precrack	1.8	load-decrease	2.8	9.7
**SENB-4**	RT	0.1	precrack	1.8	load-increase	2.6	6.2
					load-decrease	2.2	8.3
**SENB-5**	650	0.1	precrack	1.8	load-decrease	2.3	8.1
					load-increase	2.5	9.0

## References

[1] Bewlay B.P., Weimer M., Kelly T., Suzuki A., Subramanian P. (2013). The Science, Technology, and Implementation of TiAl Alloys in Commercial Aircraft Engines. MRS Proc..

[2] Edwards T. (2018). Recent progress in the high-cycle fatigue behaviour of γ-TiAl alloys. Mater. Sci. Technol..

[3] Gnanamoorthy R., Mutoh Y., Mizuhara Y. (1996). Fatigue crack growth behavior of equiaxed, duplex and lamellar microstructure γ-base titanium aluminides. Intermetallics.

[4] Hénaff G., Bittar B., Mabru C., Petit J., Bowen P. (1996). Fatigue crack propagation resistance of a Ti48Al_2_Mn_2_Nb alloy in the as-cast condition. Mater. Sci. Eng. A.

[5] Chan K.S., Shih D.S. (1997). Fatigue and fracture behavior of a fine-grained lamellar TiAl alloy. Met. Mater. Trans. A.

[6] Mercer C., Lou J., Soboyejo W. (2000). An investigation of fatigue crack growth in a cast lamellar Ti_48_Al_2_Cr_2_Nb alloy. Mater. Sci. Eng. A.

[7] Pippan R., Hageneder P., Knabl W., Clemens H., Hebesberger T., Tabernig B. (2001). Fatigue threshold and crack propagation in gamma-TiAl sheets. Intermetallics.

[8] Hamada S., Nozue A., Tamin M.N. (2000). Fatigue Crack Growth Mechanisms Of Cast Ti-48Al (at.%) Alloy. WIT Trans. Eng. Sci..

[9] Balsone S.J., Larsen J.M., Maxwell D.C., Jones J.W. (1995). Effects of microstructure and temperature on fatigue crack growth in the TiAl alloy Ti-46.5Al-3Nb-2Cr-0.2W. Mater. Sci. Eng. A.

[10] Campbell J.P., Rao K.T.V., Ritchie R.O. (1999). The effect of microstructure on fracture toughness and fatigue crack growth behavior in gamma-titanium aluminide based intermetallics. Metall. Mater. Trans. A.

[11] Gloanec A.-L., Hénaff G., Bertheau D., Belaygue P., Grange M. (2003). Fatigue crack growth behaviour of a gamma-titanium-aluminide alloy prepared by casting and powder metallurgy. Scr. Mater..

[12] McKelvey A.L., Rao K.T.V., Ritchie R.O. (2000). High-temperature fracture and fatigue-crack growth behavior of an XD gamma-based titanium aluminide intermetallic alloy. Met. Mater. Trans. A.

[13] van Kranenburg C., He W., Zuidema J., Veer F. (2004). Influence of Measurement Method on Fatigue Crack Growth Threshold. ECF15, Stockolm. https://www.gruppofrattura.it/ocs/index.php/esis/ECF15/paper/download/8725/4790.

[14] Forth S.C., Newman J.C., Forman R.G. Generating fatigue crack growth thresholds with constant amplitude loads. Proceedings of the 8th International Fatigue Congress.

[15] Suresh S. (1998). Fatigue of Materials.

[16] ASTM (1999). Standard Test Method for Measurement of Fatigue Crack Growth Rates.

[17] Pickard A.C., Brown C.W., Hicks M.A. (1983). The Development of Advanced Testing and Analysis Techniques Applied to Fracture Mechanics Lifing of Gas Turbine Components. International Conference on Advances in Life Prediction Methods.

[18] Hu D., Yan L., Gao Y., Mao J., Wang R. (2019). Crack Growth Behavior of Full-Scale Turbine Attachment Under Combined High and Low Cycle Fatigue. J. Eng. Gas Turbines Power.

[19] International ASTM (2001). Standard Test Method for Measurement of Fracture Toughness.

[20] Hussain K. (1997). Short fatigue crack behaviour and analytical models: A review. Eng. Fract. Mech..

[21] Kruzic J.J., Campbell J.P., Ritchie R.O. (1999). On the fatigue behavior of gamma-based titanium aluminides: Role of small cracks. Acta Mater..

[22] Ritchie R.O., Peters J.O. (2001). Small Fatigue Cracks: Mechanics, Mechanisms and Engineering Applications. Mater. Trans..

[23] Gloanec A.L. (2003). Mécanismes gouvernant le comportement cyclique et la résistance à la fissuration par fatigue des alliages TiAl. Ph.D. Thesis.

[24] Zhao L., Tong J., Byrne J. (2001). Stress intensity factor K and the elastic T-stress for corner cracks. Int. J. Fract..

[25] Forth S., Newman J., Forman R. (2003). On generating fatigue crack growth thresholds. Int. J. Fatigue.

[26] Newman J., Riddell W., Piascik R. (2000). Effects of K max on Fatigue Crack Growth Threshold in Aluminum Alloys. Fatigue Crack Growth Thresholds, Endurance Limits, and Design.

[27] Mall S., Staubs E.A., Nicholas T. (1990). Investigation of Creep/Fatigue Interaction on Crack Growth in a Titanium Aluminide Alloy. J. Eng. Mater. Technol..

[28] Chapetti M. (2003). Fatigue propagation threshold of short cracks under constant amplitude loading. Int. J. Fatigue.

[29] Jordon J.B., Newman J.C., Xue Y., Horstemeyer M.F. Near Threshold Fatigue Crack Growth in 7075-T651 Aluminum Alloy. Proceedings of the SEM Annual Conference and Exposition on Experimental and Applied Mechanics.

[30] Newman J., Yamada Y. (2010). Compression precracking methods to generate near-threshold fatigue-crack-growth-rate data. Int. J. Fatigue.

[31] Zhou X., Hohenwarter A., Leitner T., Gänser H., Pippan R. (2015). Load history effects on fatigue crack propagation: Its effect on the R-curve for threshold. Frat. Integrità Strutt..

[32] Botvina L.R., Korsunsky A.M. On the structure of plastic and damage zones in different materials and at various scales. Proceedings of the 11th International Conference on Fracture (ICF11).

[33] Trail S.J. (1996). Fatigue of Gamma Based Titanium Aluminide Alloys. Ph.D. Thesis.

[34] Chan K.S., Shih D.S. (1998). Fundamental aspects of fatigue and fracture in a TiAl sheet alloy. Met. Mater. Trans. A.

[35] Ast J., Ghidelli M., Durst K., Göken M., Sebastiani M., Korsunsky A., Goeken M. (2019). A review of experimental approaches to fracture toughness evaluation at the micro-scale. Mater. Des..

[36] Ghidelli M., Sebastiani M., Collet C., Guillemet R. (2016). Determination of the elastic moduli and residual stresses of freestanding Au-TiW bilayer thin films by nanoindentation. Mater. Des..

